# Crushed *Capsicum chacoense* Hunz Fruits: A Food Native Resource of Paraguay with Antioxidant and Anthelmintic Activity

**DOI:** 10.1155/2022/1512505

**Published:** 2022-03-31

**Authors:** Eva Coronel, Laura Mereles, Silvia Caballero, Nelson Alvarenga

**Affiliations:** ^1^Food Biochemistry Department, Facultad de Ciencias Químicas, Universidad Nacional de Asunción Campus Universitario, Ruta Mcal. Estigarribia Km 9,5, PO Box 1055, San Lorenzo, Paraguay; ^2^Phytochemistry Department, Facultad de Ciencias Químicas, Universidad Nacional de Asunción Campus Universitario, Ruta Mcal. Estigarribia Km 9,5, PO Box 1055, San Lorenzo, Paraguay

## Abstract

The nutritional composition and toxicity of native plants with food potential like *Capsicum chacoense* are important for the safe use of populations and could be used as a source for searching for new drug candidates. Infections produced by parasites such as helminths are a public health concern for many countries. The drugs used for treating these diseases are few, and the emergence of resistance is a risk. In this work, the nutritional composition, acute toxicity, antioxidant activity, and anthelmintic activity of crushed *C. chacoense* fruits were evaluated. The composition was analyzed by standard procedures. Antioxidant activity was evaluated using the ABTS radical and the total phenolic compound (TPC) tests. The toxicity was evaluated on Swiss albino mice by the single-DL50-dose procedure. The anthelmintic activity was tested against *Eisenia foetida*. The samples presented high levels of dietary fiber (47.05-49.19 g/100 g), proteins (14.43-15.60 g/100 g), and potassium (1708-1733 mg/100 g). In the samples, the absence of acute lethal effects in doses lower than 2000 mg/kg was observed. A rich composition of TPC (517.26-543.32 mg GAE/100 g sample), total carotenoids (125.72-239.57 mg/kg), *β*-carotene (3.29-5.60 mg/kg), and good TAC was observed (154-158 mM TEAC/g SMTC). The methanolic extracts at the doses tested (2.5 to 40 mg/mL) showed good anthelmintic activity. The presence of alkaloids was demonstrated in the methanolic extract, consistent with the levels of capsaicin (131.85 and 98.80 mg/100 g) and dihydrocapsaicin (80.75 and 63.68 mg/100 g), with significant statistical differences between samples (*p* < 0.05). These results show that through the chemical composition of this underutilized native resource and good fruit processing procedures, the *C. chacoense* fruits have a great nutraceutical potential of interest for the food and pharmaceutical industries.

## 1. Introduction

In the Chaco Central region of Paraguay, the native fruits are important food resources of the indigenous and rural communities [1]. *Capsicum chacoense* is an endemic species of this area that grows wild in hills and forests. The plant is a highly branched shrub; the fruits are globose red berries [2, 3]. The species constitutes a condiment for the Énxet, Ayoreo, and Tapiete ethnic groups, who traditionally process it, adding a characteristic so-called “Chaco flavor” to their meals [4, 5].

Due to their importance in agroeconomic, biological, and cultural terms, *Capsicum* species are cultivated throughout the world in warm and temperate regions [6]. It has been found that fresh and dry samples contain a good amount of carotenoids, being capsanthin, a characteristic carotenoid of the *Capsicum* species the major compound [7]. The good bioavailability of eight carotenoids has been verified, with capsanthin having a bioavailability of up to 97% [8]. Previous works on the fruits of *Capsicum chacoense* were carried out in *ex situ* experimental crops, far from its natural appearance.

Free radicals are implicated as one of the probable causes of cancer, aging, and certain neurodegenerative diseases. For this reason, finding substances that protect against the action of these radicals is also of great importance. Phenolic compounds as well as flavonoids and carotenoids possess antioxidant activity and have been identified in the *Capsicum* genus, making these plants a source of antioxidant substances [9–11]. These fruits have good antioxidant activity by *in vitro* total antioxidant capacity (TAC) assays, total phenol compounds (TPC), flavonoids, capsaicin, and dihydrocapsaicin contents [12].

The composition of products derived from native fruits that grow in the Chaco region, in adverse climatic conditions, low rainfall, and saline soil, is very limited, so their nutritional and nutraceutical properties are unknown in their habitual consumption form, as well as their potential applications. On the other hand, it was reported that *Capsicum chacoense* is naturally polymorphic for the production of capsaicinoids and this species shows geographic variation in the capsaicinoid proportion of individual plants, and this variation is directly related to the variation in the damage caused by a pathogenic fungus of its seeds [13].

On the other hand, with the advancement of science, more and more unknown attributes of natural products are discovered that improve the quality of life. Currently, natural products are widely used for various purposes such as for functional, antioxidant [14], and antifungal [15] properties and even for catalysis of waste products [16, 17].

Plants are a good alternative to find active substances against various illnesses that afflict mankind. Between those diseases that are a public health concern, important groups are the ones produced by helminths. A large number of people in developing countries are affected by soil helminthiasis, mainly those living in poverty, having precarious sanitary facilities, and having difficult access to fresh water supplies [18]. According to the OMS, approximately two billion people are infected by helminths around the globe. The treatment implemented is based on the use of the benzimidazole drug class (albendazole and mebendazole) that have some known adverse effects and with the risk of parasite resistance always present. With those facts, searching for new drug candidates from plants seems to be a plausible alternative [19]. On the *Capsicum* genus, anthelmintic activity was described for the methanolic extract of leaves of *Capsicum frutescens*, indicating that the genus has the potential to yield compounds that possess that kind of activity [20].

Lack of toxicity is also an important factor to assure the safety of the foods used by the populations. *Capsicum* has been studied previously, but, to the best of our knowledge, no studies have been made of the fruits processed traditionally by the native communities of the Paraguayan Chaco region; neither its anthelmintic and antioxidant activity nor its toxicity has been determined. To value a food resource from the Paraguayan Chaco and evaluate its potential applications as a nutraceutical and in pharmacy, in this work, we studied the nutritional composition, toxicity, in vitro antioxidant capacity, and anthelmintic activity of crushed C. chacoense Hunz fruits from Paraguay.

## 2. Materials and Methods

### 2.1. Plant Material

Wild *C. chacoense* fruits were collected in March 2019 at Alto Paraguay Department, from two arboreal populations. Plant materials taken from every site was randomly collected from different trees, named as sample “Estancia 1” (GPS S -21° 08,814; W -59° 25,879) and “Estancia 2” (S -21° 14,360; W -59° 33,455). Voucher specimens were deposited in the Herbarium of the Faculty of Chemical Sciences, with the codes G. Delmás 57531 and 57530.

The sampling of the whole fruits was done manually, and they were dried for 10 days in sunlight, according to the usual procedure used by the native populations. Once dry, the fruits were crushed in a wooden mortar and transferred to appropriate containers in the laboratory. For analytical determinations, the samples were homogenized in a Severin® food processor (Sundern, Germany) and stored at -20°C.

### 2.2. Proximal Composition

The proximal composition analysis was determined by AOAC official methodologies [21] moisture (method No. 934.01), ash (method No. 940.26), dietary fiber (method No. 991.42), total lipids (method No. 970.51), and total nitrogen using the conversion factor 6.25 from nitrogen to proteins (method No. 920,152). The content of total carbohydrates and soluble sugars was determined by the Clegg anthrone method, with and without previous acid hydrolysis, respectively [22]. The results were expressed in grams/100 g on a dry basis.

### 2.3. Mineral Content

For mineral analysis such as Na, K, Ca, Mg, Fe, Cu, Zn, and Mn, the atomic absorption AOAC method 975.03 was used [21]. An AA 6300 Shimadzu (Kyoto, Japan) spectrophotometer was employed. The phosphorus content was determined by colorimetric AOAC method 935.45 [21]. For each mineral, a calibration curve was made using standard solutions (Merck, Darmstadt, Alemania). The results were expressed in milligrams/100 g on a dry basis (DB).

### 2.4. Capsaicinoid Content

To determine the content of capsaicin (CAP) and dihydrocapsaicin (DHC), an extraction procedure was carried out according to Zheng et al. [23]. Quantification was carried out by HPLC as described by Nag et al. [24]. A reversed-phase C18 column (250 cm × 4.6 mm, 5 *μ*m, 100 Å, Phenomenex, Torrance, USA) was used. The mobile phase was water (A) : acetonitrile (B) (35 : 65) *v*/*v* in isocratic mode. The pH of the water was adjusted to 3.8 with 1% glacial acetic acid (*v*/*v*). The flow rate was 1.0 mL/min. The column temperature was kept at 25°C, and the injection volume was 20 *μ*L. A PDA detector was used (SPD-M20A, Shimadzu, Kyoto, Japan), with monitoring at 230 nm. Calibration curves for both CAP and DHC were made from analytical standards (Sigma-Aldrich, Saint Louis, USA), whose concentrations ranged from 1 to 80 *μ*g/mL. Figure S1 shows the chromatogram of the standards. On the other hand, the pungency level of the samples was determined by calculations according to AOAC method No. 995.03 [21].

### 2.5. Total Antioxidant Capacity Assay

The TAC assay was performed using the ABTS•+ cationic radical discoloration test [25]. First, an ultrasound-assisted extraction was carried out with methanol: water (60 : 40) and acetone : water (70 : 30). The solution was formed 24 h before performing the assay; subsequently, it was diluted with absolute ethanol to an absorbance of 0.7 ± 0.02 at 730 nm (UV-1800, Shimadzu, Kyoto, Japan). A calibration curve of 6-hydroxy-2,5,7,8-tetramethylchroman-2-carboxylic acid (Trolox) 0-280 *μ*M was used. The results were expressed as micromolar Trolox equivalents (TEAC)/g samples. The extract inhibitory concentration 50 (IC_50_) was also determined by reaction with different extract concentrations. The results were expressed in micrograms per milliliter.

### 2.6. Total Phenolic Compounds (TPC)

The sample was extracted 1 g with methanol : water (60 : 40) and then with acetone : water (70 : 30) in an ultrasonic bath for 15 min. The extracts obtained were centrifuged after each process (15 min, 4°C, 5000 rpm), and the supernatants combined. Total phenolic compounds were measured spectrophotometrically using the Folin-Ciocalteu reagent by the method described by Singleton and Rossi [26], where the blue-colored complex was quantified at 765 nm (UV-1800, Shimadzu, Kyoto, Japan). A gallic acid calibration curve (10-160 *μ*g/mL) was used. The results were expressed in milligrams of gallic acid equivalents (GAE) per 100 g of sample (mg of GAE/100 g).

### 2.7. Content of Total Carotenoids and *β*-Carotene

For the extraction of total carotenoids, the method previously described by Procisur, IICA, was used [27] and the quantification was carried out according to Sadasivam and Manikkam [28]. The extract was measured at 460 nm in the spectrophotometer, and a calibration curve for *β*-carotene of 1-10 *μ*g/mL was performed. The results were expressed in milligrams per kilogram on a dry basis.

The content of *β*-carotene was determined by HPLC-PDA with some modifications [27]. First, the extraction with BHT (in acetone) was performed. The injections were made immediately after each extraction. The chromatographic system used a C18 column (Phenomenex Inc., USA) 250 cm × 4.6 mm, 5 *μ*m, 100 Å; the oven was kept at 30°C, mobile phase : methanol : acetonitrile : triethylamine (900 : 100 : 1), premixed; pump system was in isocratic mode. The flow was 1.5 mL/min, and injection volume 20 *μ*L. The detector was PDA SPD-M20A (Shimadzu, Kyoto, Japan) at 450 nm. A calibration curve of *β*-carotene dissolved in HPLC grade acetone was used (0.3-3 *μ*g/mL). Figure S2 shows the chromatogram of the standard.

### 2.8. Preparation of the Methanolic Extract

To obtain the methanolic extract, 750 mL of HPLC grade methanol was used for every 100 g of finely ground sample. The extraction was assisted by ultrasound for 30 min; then, it was allowed to stand for 15 minutes, and the procedure was repeated 3 times. Subsequently, the material was left in contact with the solvent, overnight. Once the sample was filtered, the procedure was repeated 2 times. Then, the residue was extracted with the same solvent by refluxing 15 min, repeating the procedure 2 times with changes in the solvent. All individual extracts obtained were mixed and concentrated in a vacuum rotary evaporator at 40°C.

### 2.9. Phytochemical Profile

Qualitative tests were carried out from the methanolic extract using precipitation and coloration reactions along with thin-layer chromatography (TLC) as previously described [29]. Secondary metabolites such as alkaloids, steroids, triterpenes, flavonoids, tannins, naphthoquinones and anthraquinones, saponins, leukoanthocyanidins, cardiotonics, terpenes (terpenic lactones), coumarins, cardenolides, and *α*-*β* unsaturated lactones were determined. The results were expressed as little (+), moderate (++), or abundant (+++), respectively, according to the amount of the precipitate or the intensity of the coloration obtained.

### 2.10. Acute Toxicity

The assay was performed according to the Fixed-Dose Procedure (FDP) proposed by the British Toxicology Society Working Party on Toxicity [30] and currently included in OECD Guide 420 [31], using oral administration, and complied with the ARRIVE guidelines, by the U.K. Animals (Scientific Procedures) Act 1986 and associated guidelines and EU Directive 2010/63/EU for animal experiments; Swiss albino female mice weighing 25-30 g were used, which were fasted before carrying out the test. The extracts were dissolved in water : propylene glycol : ethanol (50 : 40 : 10) with the help of an ultrasonic bath. The doses used were 500, 1000, and 2000 mg/kg of body weight, and the blank used was the solvent of the extracts. Animals were observed for behavioral changes during the first two hours and for lethality during the first 24 hours and periodically for 14 days. After 14 days of observation, the mice were sacrificed by cervical dislocation and internal organs (stomach, intestines, kidney, liver, are spleen) were analyzed for abnormalities.

### 2.11. Anthelminthic Activity

The methanolic extract was tested for anthelmintic activity according to a procedure described [32]. The model used was *Eisenia foetida* or Californian red worm (4-6 cm long). The extract concentrations were 2.5, 5, 10, 20, and 40 mg/mL. For each concentration, three worms of approximately the same size and diameter were distributed in a Petri dish. All the trials were made in triplicate and repeated on three different days. Albendazole (10 mg/mL) and DMSO in sterile saline as the positive and negative control, respectively, were used. “Time to paralysis” and “time to death” (in minutes) were the variables. To determine if the worms were dead, they were put in contact with water at 50°C, where lack of movement indicated death, and the corresponding time was recorded.

### 2.12. Ethical Considerations

The protocol was approved by the Research Ethics Committee of the Facultad de Ciencias Químicas (CEI code 481/19). Work was done respecting the standards established by the Ethics Commission of the European Community [33], for the handling of laboratory animals. The principle of the 3Rs or Alternatives in the use of Laboratory Animals (Replacement, Reduction, and Refinement) was applied.

### 2.13. Statistical Analyses

The data were recorded and processed in the GraphPad Prism 5.0 program (GraphPad Software Inc., CA, USA). Descriptive statistics were applied. To determine if significant differences between samples existed, the Student *t*-test (*p* ≤ 0.05) was used. For the bioassays, one-way ANOVA with Tukey posttest at a confidence level of 95% was used for the comparisons between groups. Analyses were conducted at least three times with three different samples. Each experimental value is expressed as the mean ± standard deviation (SD).

## 3. Results and Discussion

### 3.1. Nutritional Composition

**Figure 1:**
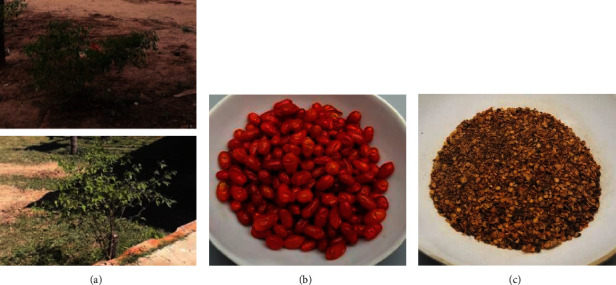
*Capsicum chacoense* (a) wild plant on Chaco Central, (b) fresh fruits, and (c) crushed dried samples.

The centesimal composition of the samples (Figure 1) is presented in Table 1. Statistically significant differences were observed (Student's *t*-test, *p* ≤ 0.05) between samples, except in the content of total carbohydrates, soluble sugars, dietary fiber, and potassium. Low moisture was observed in both samples (less than 10%), as reported by other authors [34, 35] for another species of dried *Capsicum* sp fruits. It is noteworthy that the analyzed samples had already been subjected to a drying process in the sun before reaching the laboratory, a method used by the residents for food consumption, so the results of the analyzed samples are not comparable with fresh samples freshly harvested, as described in other *Capsicum* species [36, 37]. Dietary fiber was the major component; data on the centesimal or proximal composition of *C. chacoense* is scarce in the literature; however, studies have been carried out in species of the same genus, such as in fruits of *C. annuum* grown in India, where 18.98 g/100 g of crude fiber has been reported [35]; the highest value observed in this work may be because the seeds have been included since these are the majority of the small berries of *C. chacoense*, which agrees with that reported by Zou et al. The protein values were similar to the protein content of *C. chinense* (16.37 g/100 g) [39]; both lower values (11.7 g/100 g) have been reported in *C. frutescens* [40] as higher values in *C. annuum* (17-18 g/100 g) [35, 36] to those of this work. Regarding the mineral content of the samples, the potassium content among the macronutrients and iron among the micronutrients stood out (Table 1). The most abundant mineral in the samples was K in both samples without showing statistically significant differences between means (Student\s *t*-test, *p* ≤ 0.05).

Higher K values have been described in *C. annuum* [41], as well as lower values for the same [38, 42, 43]. Like most plant foods, *C. chacoense* had a low sodium content despite the salty soil of Chaco; statistically significant differences were observed between samples from different sampling sites (Student test, *p* ≤ 0.05). The sample's K/Na ratios are also higher. Potassium is the most abundant mineral in vegetables; however, the relationship commonly found in vegetables is 9 times more K than Na; the Na values observed were higher than those described for other species of the genus *Capsicum* [38, 41, 43], and 25 times more K than Na was found. The Solanaceae family as well as that of the Capsicum genus is of great interest to advance the production of resistant crops from their wild relatives, which could be exploited to contribute to food security over the coming decades. Creating crops with resistance to drought, soil salinity that simultaneously has higher nutritional quality, is challenging to conventional breeding. Advances in gene-editing technology, coupled with analysis of crop “pangenomes,” may allow rapid conversion of crop wild relatives into crops, retaining valuable resilience and nutritional [44]. *C. chacoense* offers a genetic base to advance in this line, due to the saline soil of its wild appearance.

### 3.2. Capsaicinoid Content and Level of Pungency

Alkaloid compounds such as capsaicin and dihydrocapsaicin and the level of pungency in the sample were studied (Table 1), and a higher content of capsaicin than dihydrocapsaicin was observed. Significant differences are observed within samples (Student's *t*-test, *p* ≤ 0.05), the highest values on Estancia 1 sample. The capsaicin content found is ten times higher than that reported by López et al. in *C. chacoense* fruits grown in Salta, Argentina (13.9 mg/100 g of dried fruit), which may be due to differences in the extraction process (dichloromethane at 80°C and methanol : water 70 : 30 for 24 h cold) [45]. On the other hand, in Italy, Loizzo et al. analyzed the capsaicin content in fresh, boiled, and frozen *Capsicum* fruits from controlled condition crops, where *C. chacoense* presented higher levels than those observed in this work for the dried fruits. It should be noted that a limitation of this work was that there was no access to fresh fruits and that the analyzed samples were subjected to postharvest treatments (traditional) such as drying in the sun and grinding in wooden mortars; however, it was analyzed in the conditions such as the wild fruits of this species which are commonly used.

Regarding the content of the alkaloid dihydrocapsaicin (Table 1), values of 50 to 60 mg/100 g of dihydrocapsaicin have been reported in fresh, frozen, and boiled fruits of *Capsicum* [12]. This range is lower than that observed in the present work for nuts, where it is expected that drying will concentrate the components of the sample due to its low water content; however, the difference observed with the fresh results is not very striking. In America, for other species of *Capsicum*, cultivated in Peru, of 32 species of *C. pubescens*, the variation observed in the content of dihydrocapsaicin was from 25 to 207 mg/100 g of dried fruit [46].

According to the categorization proposed by Eich, capsaicinoids can characterize food pungency as mild (0–5,000 SHU), medium (5,000–20,000 SHU), hot or spicy (20,000–70,000 SHU), and extremely hot or spicy (70,000–300,000 SHU) [47]. The analyzed samples are in the “spicy” category, according to what is reported in Table 1. The *C. chinense*, variety “Carolina reaper,” since 2017 is considered the hottest chili pepper in the world according to the Guinness Book of Record with 1,641,183 SHU [48], a value much higher than that observed in this work for *C. chacoense*. However, there are other varieties of *C. chinense* that are also in the spicy category (62,000 SHU) [49]. On the other hand, it has been reported that the varieties of *C. annuum* have a lower level of pungency (362.5 to 9720 SHU) compared to the samples of *C. chacoense* analyzed [50, 51].

Capsaicin and dihydrocapsaicin content, along with volatile organic compound (VOC) emission comparison and proteomic profile methods, allows comparison of compositional characteristics with a more precise approach concerning traditional variety descriptors. The identification of putative volatiles is a good candidate for varietal recognition and could be addressed in future studies to assess the diversity of these wild food resources. For example, protein profiles in the leaves have been reported to allow this differentiation, and the wild species *C. chacoense* has been shown to have more similarity to the domesticated pepper *C. annuum*, compared to *C. baccatum* [6]. On other hand, the level of pungency observed on *C. chacoense* makes it appropriate for uses as a flavoring in food, in addition to other uses that can be given to substances with these characteristics, such as for pharmacological uses in the preparation of topical or industrial creams and the manufacture of personal protection weapons.

### 3.3. Phytochemical Composition

In the phytochemical profile, the abundant presence of alkaloids, as well as the presence of tannins, and to a lesser extent free steroids and/or triterpenoids, flavonoids, and saponins was observed (Table 2). Naphthoquinones and/or anthraquinones, coumarins, cardiotonics, and terpenic lactones were not observed.

Alkaloids were the most abundant secondary metabolites, as expected from the known presence of capsaicinoids in this genus. In addition, the presence of tannins, another characteristic component of the *Capsicum* species, was also observed. In fruits of *C. annuum*, the presence of compounds from this group such as gallic acid, caffeic acid, luteolin, vanillic acid, ferulic acid, and various compounds derived from them has been reported [52]. There are references to the presence of flavonoids in *C. chacoense* that validate the positive results observed in the cyanidin and HCl reaction. In experimental cultures, the content of total flavonoids was 0.5-1.5 mg/100 g, one of the highest among species cultivated [12]. In America, the presence of flavonoids such as quercetin and luteolin has been described in *C. annuum*, *C. chinense*, and *C. baccatum* [53].

When performing two-dimensional TLC and developing it with the Liebermann-Burchard reagent, lilac spots were observed, which is indicative of the presence of free steroids and/or triterpenoids. With the foam test, a positive result was obtained for the presence of saponins in the extracts studied. The presence of saponins in the fruits of *C. baccatum* has been reported by the same method used in this work, in addition to the hemolysis test [54]. Saponins have shown important antifungal activities against various fungi [55, 56].

### 3.4. Antioxidant Potential

The antioxidant potential of crushed *C. chacoense* fruits was measured based on the total antioxidant capacity (inhibition of the radical ABTS•+), the content of total phenols, total carotenoids, and *β*-carotene as summarized in Table 3. Significant differences were observed (Student's *t*-test, *p* ≤ 0.05) between both samples about the content of total carotenoids and *β*-carotene.

Regarding the values of the IC_50_, it was observed that the methanolic extract of the samples presented lower antioxidant activity than Trolox (139 mM).

Values of extracts reported in *Capsicum* species in the fresh state are lower than what was found in this work [12]. Related to the equivalent values of Trolox, other authors have reported that in different species and varieties of controlled *Capsicum* cultivars, the antioxidant capacity of the extracts ranged from 180 to 920 mM TEAC/g in the samples of *C. chinense*, *C. frutescens*, *C. baccatum*, *C. annuum*, and *C. pubescens* [46, 53]. Thus, higher values of total phenols have also been reported than those found in this work for fresh, frozen, and boiled fruits of *C. chacoense* [12]; and in comparison with other species of *Capsicum*, both higher and lower values of total phenols have been described in fruits that had different treatments [12, 52]. These differences are clearly explained by postharvest treatments, crop conditions, genetics, and environmental factors mentioned above. Future studies on the optimal conditions of cultivation, drying, storage, and extraction of bioactive components of *C. chacoense* are necessary to take advantage of these properties in this type of fruit at the local level.

Regarding the content of total carotenoids, it was observed that the Estancia 2 sample had a higher level than the Estancia 1 sample (Table 3). In a study carried out in Mexico, they found that the total carotenoid content in fresh whole fruits of *C. chacoense*, from controlled crops, was 633 mg/kg [57], higher than that observed in this work. In the literature, a wide range of values for carotenoids is reported in different *Capsicum* species such as *C. annuum*, *C. baccatum*, *C. chinense*, *C. frutescens*, and *C. annuum* [35], where the values observed in this work are within the range reported for other *Capsicum* species. Regarding the content of *β*-carotene, different authors report a higher content of *β*-carotene in fruits of *C. chacoense* (137 mg/kg to 36,660 mg/kg) [7, 8, 57] to those of this work. The observed *β*-carotene content represented 2.3-2.6% of the total carotenoids; however, it can be observed that the samples presented other peaks in the carotenoid chromatogram (see Figure S3) which have not been identified and quantified in this work in the absence of reference standards. Within the carotenoid profile of *C. chacoense*, compounds such as violaxanthin, neoxanthin, antheraxanthin, lutein, capsanthin, zeaxanthin, *β*-cryptoxanthin, and *β*-carotene have been described [49, 50], being capsanthin, a carotenoid of the genus *Capsicum* the majority. Other studies are considered necessary to identify the carotenoids present in these “ají del monte” fruits since the carotenoid content gives an added commercial value and the *Capsicum* resins are used as natural colorants in the food industry [58].

According to these results, the antioxidant potential of the samples of *C. chacoense* from nuts is lower than that described for the same species by other authors in terms of its content of polyphenols, carotenoids, and total antioxidant activity by ABTS. The main factor of these variations, in addition to the genetics and environmental conditions of growth, could be the postharvest treatment used by the inhabitants for the fruits of *C. chacoense*.

### 3.5. Acute Toxicity

No signs of toxicity were observed when performing the Fixed-Dose Procedure test in samples of the methanolic extract of the *C. chacoense* sample. Also, abrupt changes in behavior up to a dose of 2000 mg/kg were not observed. The animals used in the test survived in good health, without macroscopic changes in the organs during the 14 days that the test lasted. In the necropsy, no macroscopic differences were observed in the vital organs concerning the control in the stomach, small intestine, large intestine, liver, kidney, spleen, heart, and lung. These results showed that the methanolic extracts of the crushed *C. chacoense* fruits are devoiced of acute toxicity effects in the test conditions.

### 3.6. Anthelmintic Activity *In Vitro*

**Figure 2:**
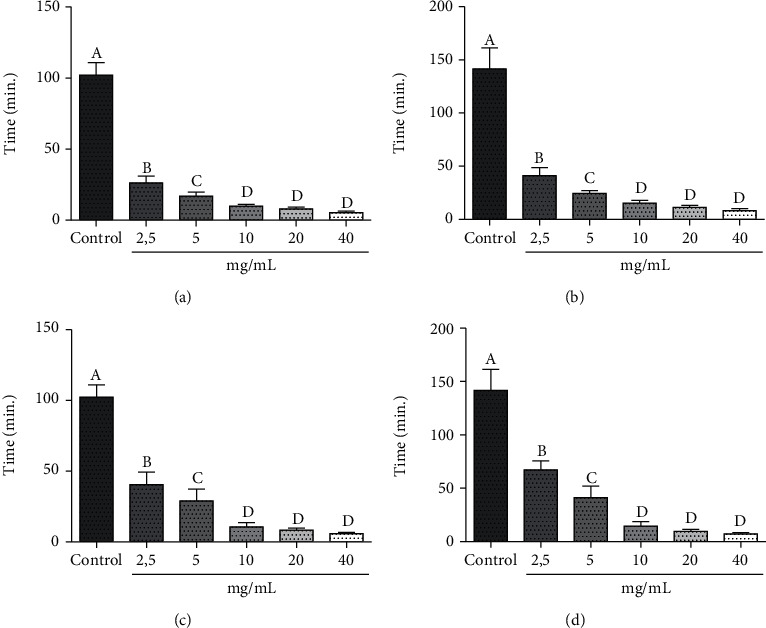
*In vitro* anthelmintic activity of the methanolic extract of crushed *Capsicum chacoense* fruits with *Eisenia foetida*. (a) Paralysis time for the methanolic extract of the Estancia 1 sample. (b) Time of death for the methanolic extract of the Estancia 1 sample. (c) Paralysis time for the methanolic extract of the Estancia 2 sample. (d) Time of death for the methanolic extract of the Estancia 2 sample. Data represented as mean ± SD. Different lowercase letters between columns indicate significant differences between means, ANOVA, Tukey's *post hoc* test (*p* ≤ 0.05). Positive control: albendazole 10 mg/mL. Negative control: DMSO+physiological solution.

The anthelmintic activity *in vitro* of the methanolic extract of the crushed *C. chacoense* fruits was evaluated using the *Eisenia foetida* model (Figure 2). In all cases, the paralysis and death timeswere lower than the positive control (albendazole, 10 mg/mL), with statistically significant differences between the control and the extracts (ANOVA, Tukey's a posteriori test, *p* ≤ 0.05). These results indicate that the extracts had better anthelmintic activity in vitro than albendazole, which could be due to the high content of capsaicinoids in the analyzed samples.

It should be noted that at concentrations greater than 10 mg/mL, no significant differences were observed between the extracts; however, among the concentrations of 2.5 and 5 mg/mL, significant differences were observed, which may point that the potential therapeutic range of these extracts is at concentrations lower than 10 mg/mL. Therefore, subsequent work should be carried out for the identification of the metabolite (s) responsible for the anthelmintic activity observed in the samples. On the other hand, the paralysis and death times of the extract obtained from the Estancia sample (5.80-26.27 min and 8.26-41.21 min, respectively) were longer than that of the Estancia 2 sample (10.63-40, 43, and 7.28-67.19 min, respectively).

Anthelmintic activity has also been reported in the methanolic extract of *C. frutescens* fruits in worms of the *Tubifex tubifex* species, where they found that the paralysis time varied from 18.28 to 3.43 and the death time from 37.92 to 5.89 for concentrations of 2.5 to 10 mg/mL [59]. The leaves of *C. frutescens* have also been reported to have anthelmintic activity compared to another model of *Pheretima posthuma* in concentrations ranging from 10 to 100 mg/mL, obtaining values much higher than those of this study, where the paralysis time varied from 110.42 to 198.35 min, and the time of death ranged from 151.09 to 256.23 min [20]. These differences may be due to the biological variability of both the species studied and the species of the worm used.

## 4. Conclusions

The crushed *C. chacoense* fruits analyzed, of wild origin that was subjected to traditional postharvest treatment, have a high content of dietary fiber, possibly due to a large number of seeds and scarce amount of pulp that the berries have, in addition to the K/Na, so they are low in Na. The *ají del monte* fruit has great potential in the food industry as a flavoring due to its capsaicinoid content and coloring due to its high content of carotenoids. On the other hand, it was also demonstrated that the methanol extracts of the samples analyzed have anthelmintic activity that could be attributed to the capsaicinoid alkaloids since these are the main secondary metabolites.

## Figures and Tables

**Table 1:** Chemical composition of crushed *Capsicum chacoense* Hunz fruits.

Components	Estancia 1	Estancia 2
Moisture (g/100 g)	8.62 ± 0.07^a^	9.70 ± 0.02^b^
Lipids (g/100 g)	6.46 ± 0.03^a^	10.42 ± 0.37^b^
Ash (g/100 g)	7.07 ± 0.06^a^	5.50 ± 0.15^b^
Proteins (g/100 g)	15.60 ± 0.46^a^	14.43 ± 0.51^b^
Total carbohydrates (g/100 g)	11.35 ± 1.46^a^	11.41 ± 0.66^a^
Soluble sugars (g/100 g)	3.46 ± 0.10^a^	3.37 ± 0.17^a^
Dietary fiber (g/100 g)	49.19 ± 1.08^a^	47.05 ± 0.31^a^
Sodium (mg/100 g)	69.17 ± 3.50^a^	40.08 ± 4.15^b^
Potassium (mg/100 g)	1707.91 ± 21.08^a^	1733.26 ± 37.91^a^
Calcium (mg/100 g)	190.92 ± 6.41^a^	138.44 ± 3.15^b^
Magnesium (mg/100 g)	137.67 ± 8.27^a^	175.46 ± 4.55^b^
Phosphorus (mg/100 g)	326.87 ± 2.93^a^	309.08 ± 0.26^b^
Iron (mg/100 g)	3.04 ± 0.05^a^	2.33 ± 0.09^b^
Zinc (mg/100 g)	1.77 ± 0.10^a^	1.72 ± 0.41^b^
Copper (mg/100 g)	0.57 ± 0.02^a^	0.61 ± 0.07^b^
Manganese (mg/100 g)	0.78 ± 0.03^a^	0.62 ± 0.04^b^
Calories (kcal/100 g)	234	219
Capsaicin (mg/100 g)	131.85^a^	98.80^b^
Dihydrocapsaicin (mg/100 g)	80.75^a^	63.68^b^
Pungency (SHU)	31890^a^	24372^b^

Values are expressed as mean ± standard deviation (*n* = 3). Different lowercase letters in each row indicate a significant difference between the means, Student's *t*-test (*p* ≤ 0.05).

**Table 2:** Phytochemical profile of the methanolic extract of crushed *Capsicum chacoense* Hunz fruits.

Compounds	Tests performed	Estancia 1	Estancia 2
Alkaloids	Dragendorff	(+++)	(+++)
	Mayer	(+++)	(+++)
	Valser	(+++)	(+++)
	Ammonium reineckate	(-)	(-)
Free steroids and/or triterpenoids	Two-dimensional TLC	(+)	
	Developer: Liebermann-Burchard		(+)
Flavonoids	Cyanidin reaction	(+)	(+)
	Reaction with HCl	(+)	(+)
Naphthoquinones and/or anthraquinones	Borntrager-Krauss reaction	(-)	(-)
Tannins	Gelatin-salt, FeCl3	(++)	(++)
Saponins	Foam	(+)	(+)
Coumarins	TLC	(-)	(-)
	Developer: Ferric Hydroxamate reaction		
Cardiotonic	TLC	(-)	(-)
	Developer: Raymond reaction		
Terpenic lactones	TLC	(-)	(-)
	Developer: vanillin-o-phosphoric acid		

The identification comprised staining and/or precipitation reactions along with thin-layer chromatography for the detection of steroids, triterpenes, and terpenic lactones. +++: abundant; ++: moderate; +: scarce; -: absent.

**Table 3:** Antioxidant potential of crushed *Capsicum chacoense* Hunz fruits.

	Unit of measurement	Estancia 1	Estancia 2
Antioxidant capacity (ABTS+)	IC50 (*μ*g/mL)	1055 ± 122^a^	1090 ± 57^a^
	mM TEAC/g SMTC	154 ± 5^a^	158 ± 4^a^
Total phenols	mg GAE/100 g sample	543.32 ± 7.30^a^	517.26 ± 28.92^a^
Total carotenoids	mg/kg of sample	125.72 ± 6.26^a^	239.57 ± 12.39^b^
*β*-Carotene	mg/kg of sample	3.29 ± 0.03^a^	5.60 ± 0.49^b^

Data represented as mean ± SD. Different lowercase letters in each row indicate statistically significant differences between the means (Student's *t*-test, *p* ≤ 0.05).

## Data Availability

The data used to support the findings of this study are available from the corresponding author upon request.

## Supplementary Materials

Supplementary Materials Figure S1: chromatogram of the *β*-carotene standard at 3 *μ*g/mL. Figure S2: chromatogram of standard solutions of capsaicin (a) and dihydrocapsaicin (b) at 80 *μ*g/mL. Figure S3: carotenoid chromatogram of crushed *Capsicum chacoense* fruits.
