# Supplementary material for: Crushed Capsicum chacoense Hunz Fruits: A Food Native Resource of Paraguay with Antioxidant and Anthelmintic Activity
Source: Int J Food Sci. 2022 Mar 31;2022:1512505. doi: 10.1155/2022/1512505 (PMC8993580; doi:10.1155/2022/1512505)
Supplement: Supplementary Materials — Figure S1: chromatogram of the β-carotene standard at 3 μg/mL. Figure S2: chromatogram of standard solutions of capsaicin (a) and dihydrocapsaicin (b) at 80 μg/mL. Figure S3: carotenoid chromatogram of crushed Capsicum chacoense fruits. [file 1512505.f1.docx]

## Supplementary Materials

Figure S1. Chromatogram of the β-carotene standard at 3 μg/mL.

Figure S2. Chromatogram of standard solutions of capsaicin (a) and dihydrocapsaicin (b) at 80 μg/mL.

Figure S3. Carotenoid chromatogram of crushed *Capsicum chacoense* fruits.
